# Whose Expertise Is It? Evidence for Autistic Adults as Critical Autism Experts

**DOI:** 10.3389/fpsyg.2017.00438

**Published:** 2017-03-28

**Authors:** Kristen Gillespie-Lynch, Steven K. Kapp, Patricia J. Brooks, Jonathan Pickens, Ben Schwartzman

**Affiliations:** ^1^Psychology, College of Staten Island and The Graduate Center, City University of New YorkNew York, NY, USA; ^2^College of Social Sciences and International Studies, University of ExeterExeter, UK; ^3^Educational Psychology, University of California at Los AngelesLos Angeles, CA, USA

**Keywords:** autism, knowledge, stigma, neurodiversity, autistic expertise

## Abstract

Autistic and non-autistic adults’ agreement with scientific knowledge about autism, how they define autism, and their endorsement of stigmatizing conceptions of autism has not previously been examined. Using an online survey, we assessed autism knowledge and stigma among 636 adults with varied relationships to autism, including autistic people and nuclear family members. Autistic participants exhibited more scientifically based knowledge than others. They were more likely to describe autism experientially or as a neutral difference, and more often opposed the medical model. Autistic participants and family members reported lower stigma. Greater endorsement of the importance of normalizing autistic people was associated with heightened stigma. Findings suggest that autistic adults should be considered autism experts and involved as partners in autism research.

## Introduction

Traditional expert knowledge of autism derives from observations by professionals who often lack the lived experience of being autistic^1^, whose understanding and acceptance of autism might increase by listening to autistic people (Nicolaidis, 2012). Many autistic scholars and self-advocates view autism as a form of diversity rather than pathology, and an increasing number of researchers similarly conceptualize autism in terms of strengths and weaknesses rather than only deficits (e.g., Pellicano and Stears, 2011). Scientifically based knowledge of autism has tended toward greater recognition of interpersonal and developmental capacities over time, such as the reclassification of the conception that the majority of autistic individuals have intellectual disability from accurate (Stone, 1987) to false (Gillespie-Lynch et al., 2015) on the Stone Autism Awareness questionnaire, a change assisted by autistic professionals who specialize in autism, such as researcher Michelle Dawson (Dawson et al., 2007). Nevertheless, the deficit- and behavior-based diagnostic criteria for autism that anchor autism research and treatment continue to locate communication problems **within** the autistic person (American Psychiatric Association [APA], 2013) rather than examining how interpersonal interactions (De Jaegher, 2013) and societal factors (e.g., Kapp et al., 2013; Kenny et al., 2016) contribute to the challenges experienced by autistic people. The conception of autism as only an impairment within autistic people has been critiqued by autistic (e.g., Milton, 2012; Yergeau, 2013) and non-autistic (e.g., McGuire and Michalko, 2011; Dinishak and Akhtar, 2013) scholars and advocates for misrepresenting mutual challenges between autistic and non-autistic people, and facilitating misconceptions of and stigma toward autistic people.

Increasing interest in the degree to which challenges associated with autism arise from societal misconceptions about and stigma toward autism has contributed to a growing body of research examining misconceptions of and stigma toward autism among people who are **not** autistic (e.g., Gray, 1993; Mak and Kwok, 2010; Campbell and Barger, 2014; Obeid et al., 2015; Harrison et al., 2017). This research, typically conducted with non-autistic college students, has found that greater knowledge of autism and high-quality personal connections with autism coincide with lower stigma toward autism (Nevill and White, 2011; Gardiner and Iarocci, 2014; Gillespie-Lynch et al., 2015; White et al., 2016). A much smaller but growing body of research has examined how autistic people think about autism, including their evaluations of how it is currently represented and researched (e.g., Kapp et al., 2013; Pellicano et al., 2014a,b; Jones et al., 2015; Kenny et al., 2016; Fletcher-Watson et al., 2017). The current study is the first to compare the degree to which autistic and non-autistic people agree with extant scientific knowledge about autism, how they define autism, and the degree to which they endorse stigmatizing conceptions of autism. Our central hypothesis, that autistic adults would demonstrate greater awareness of scientific knowledge about autism and would describe autism in less stigmatizing ways than non-autistic people, is grounded in growing evidence that autistic people may often have enhanced understanding of fellow autistic individuals (e.g., Komeda, 2015) and resist medical constructions of autism (e.g., Kapp et al., 2013).

Evidence that autistic people often build upon unique insights derived from the lived experience of being autistic to obtain heightened knowledge about autism as a scientific construct would provide further impetus for emerging efforts to involve autistic people meaningfully in autism research and design of interventions. Recent literature reviews of participatory research about autism, wherein autistic people are meaningfully involved in all aspects of research, have revealed very few autism studies utilizing a participatory approach (Jivraj et al., 2014; Wright et al., 2014; but see Nicolaidis et al., 2011 for a strong example of participatory autism research). Indeed, autistic respondents to an online survey reported that interventions for autistic people are not well-aligned with their needs and interests because autistic people are rarely involved in designing such interventions (McLaren, 2014).

### Autistic People’s Knowledge of Autism

Failure to consider the perspectives of autistic people is problematic not only based on principles that value active participation by people in matters about their lives, but also because some autistic people use the focused interests that are part of the diagnostic criteria for autism to systematically research autism itself (e.g., Hurlbutt and Chalmers, 2002). Analyses of discussion forum posts revealed that many autistic adults followed closely, and had strong opinions about, the revision of the diagnostic criteria for autism in the DSM-5 (American Psychiatric Association [APA], 2013; Giles, 2014; Linton et al., 2014). Moreover, interviews with three autistic adults revealed that they had researched autism extensively and considered themselves “the experts” on autism (Hurlbutt and Chalmers, 2002, p. 105). Nevertheless, interviews with 11 autistic adults published a decade later revealed that participants felt that their perceptions continued to be overlooked in favor of academic expertise (Griffith et al., 2012). As one autistic participant stated, “everybody is an expert bar the person with a diagnosis. That needs to change” (p. 14).

### Autistic People Question Common Assumptions about Autism

Autistic people describe unique insights about autism derived from the lived experience of being autistic (Jones et al., 2013, 2015; Huws and Jones, 2015). They report that non-autistic people often do not understand autistic traits such as repetitive body movements, which autistic people say reflect sensory atypicalities (Davidson, 2010; Donnellan et al., 2012) and self-regulation strategies (Yergeau, 2016). Although autism is defined by socio-communicative difficulties in conjunction with restricted and repetitive interests and behaviors (RRIB), non-autistic laypeople often define autism solely in terms of socio-communicative difficulties and fail to recognize RRIB, including sensory difficulties, as core aspects of autism (e.g., Bakare et al., 2009; Campbell and Barger, 2014). In contrast, autistic people indicate that sensorimotor challenges contribute to their socio-communicative challenges (e.g., McGeer, 2004; Chamak et al., 2008; Robledo et al., 2012).

Interviews with nine autistic college students at a specialized college for autistic people revealed that they felt that only autistic people could truly understand autism (Jones et al., 2013). However, they felt that each autistic person could only speak about their own form of autism rather than about autism more generally. Participants indicated that non-autistic people have stigmatizing misconceptions about autism. They reported that sharing insider perspectives of autism with other autistic people conferred a sense of belonging. An autistic co-author of this study stated that autistic participants’ beliefs that non-autistic people could not understand autism reflected autism-related difficulties adopting other people’s perspectives. He asserted that autistic people develop the self-awareness and communication skills needed to educate others about autism with age. Indeed, additional analyses of the aforementioned interviews revealed that the autistic students felt that they had become increasingly socially aware with development (Huws and Jones, 2015).

While autism researchers often interpret neutral and even positive differences as deficits (Gernsbacher et al., 2006), autistic adults often positively reinterpret the diagnostic criteria for autism (Rosqvist, 2012a). Autistic people commonly report feeling relieved when receiving an autism diagnosis in adulthood (Punshon et al., 2009; Jones et al., 2014) and better understood by other autistic people (Sinclair, 2010; Rosqvist, 2012b; Jones et al., 2013). Similarly, interviews with 10 autistic adolescents revealed that most did not feel that autism was a disability (Jones et al., 2015). Although they recognized challenges associated with autism, such as stigma, they valued the ways that autism made them unique. Nevertheless, many autistic youth describe autism as a source of struggle or a barrier to social inclusion and yearn for normalcy (Humphrey and Lewis, 2008).

Many autistic adults identify societal factors that contribute to challenges associated with autism (e.g., Kapp et al., 2013). They embrace an identity as autistic and reject “neurotypical” behavioral norms by aligning themselves with neurodiversity, i.e., the viewpoint that autism is a natural identity with strengths and weaknesses that contribute valuably to human diversity (Hurlbutt and Chalmers, 2002; Sinclair, 2010; Nicolaidis, 2012; Rosqvist, 2012a; Walker, 2012; Kapp et al., 2013; Pellicano et al., 2014b). The neurodiversity movement challenges the medical model, wherein autism is framed as an impairment within the individual that should be treated and normalized, by emphasizing positive aspects of autism and rejecting the need to normalize autistic people and cure autism.

The neurodiversity movement has been misinterpreted as only representing the voices of “high-functioning” autistic people by researchers (Bagatell, 2010; Jaarsma and Welin, 2012) and the popular media. For example, Lutz (2013), the mother of a “lower-functioning” autistic child, wrote an article in Slate Magazine entitled “*Is the Neurodiversity Movement Misrepresenting Autism?*” She stated that the neurodiversity movement is “a group of high-functioning individuals opposed to medical research” because they themselves do not need it. However, self-advocates in the neurodiversity movement vary widely in their support needs; they often reject functioning labels as hierarchizing development in relation to an illusory ideal of “normal” that some autistic people are more (i.e., high-functioning) or less (i.e., low-functioning) close to while obscuring contextual variations in abilities, with potentially adverse consequences in terms of autistic individuals obtaining needed supports (Savarese, 2010; Yergeau, 2010; Bascom, 2012; Nicolaidis, 2012; Walker, 2012). Indeed, the neurodiversity movement and the medical model overlap in recognizing that supports are needed to ameliorate challenges associated with autism (Nicolaidis, 2012). For example, an online survey revealed that autistic adults were more likely than non-autistic people to be aware of neurodiversity and to view autism as an essential aspect of identity that needs no cure (Kapp et al., 2013). A subsequent online survey replicated Kapp et al.’s (2013) finding that autistic adults prefer terms for autism, such as “autistic person,” that indicate that autism is a central aspect of identity (Kenny et al., 2016).

#### Autistic People Recognize Challenges Associated with Autism

Despite viewing autism as central to identity, autistic participants in the study by Kapp et al. (2013) did not differ from non-autistic participants in negative emotions toward autism or in the perceived importance of supports to help autistic people gain adaptive skills. This overlap between the neurodiversity movement and the medical model indicates a more nuanced perspective on disability than the standard social model wherein impairments are believed to arise solely from societal factors. The perspective of autism endorsed by many members of the neurodiversity movement is more consistent with a biopsychosocial model (Engel, 1977) of autism, wherein internal differences interact with social factors to create challenges associated with autism (Kapp, 2013). For example, an autistic researcher pointed out that reduced theory of mind, which has been postulated to be a core deficit within autistic people (Baron-Cohen et al., 1995), is not an impairment that resides within autistic people but rather a **mutual difficulty** relating, as neurotypical people also face often unacknowledged challenges understanding the minds of autistic people (Milton, 2012). Further evidence that autistic adults’ perceptions of autism align with a biopsychosocial model arises from research demonstrating that some autistic adults recognize that autistic traits interfere with employment and socialization, and attempt to pass as “normal” (Griffith et al., 2012).

### Stigma: A Key Challenge Associated with Autism

Behaviors associated with autism may elicit higher levels of stigma than the label “autism” does (Butler and Gillis, 2011). In fact, stigma toward behaviors associated with autism is reduced when people are made aware that the people exhibiting the behaviors have a diagnosis (Brosnan and Mills, 2016), by decreasing perceptions of personal responsibility for atypical actions (Chambres et al., 2008). Indeed, autistic people with fewer symptoms (who appear more “normal”) report higher levels of stigma directed toward them than their more severely affected peers (Shtayermman, 2009), possibly because non-autistic people misinterpret them as intentionally deviant.

Stigma toward autism is reduced not only among those who are made aware that someone has a diagnosis but also among people who have more prior experience with autism. Studies conducted with college students have revealed more knowledge about (Tipton and Blacher, 2014) and less stigma (assessed with a Social Distance Scale) toward autistic people among a small number of participants who indicated that they had nuclear relatives who were autistic and/or who were autistic themselves (Gillespie-Lynch et al., 2015). Other research with college students similarly finds greater willingness to interact with (Nevill and White, 2011) or more positive attitudes toward (White et al., 2016) autistic peers among those with an autistic relative or personal contact, with further findings that the *quality* rather than quantity of direct contact plays a decisive role in acceptance of an autistic peer (Gardiner and Iarocci, 2014). White et al. (2016) reported that those who knew an autistic person less often identified observable behaviors such as lack of eye contact in connection with autism, which they suggested might indicate that with personal experience people may find that autistic individuals do not all conform to stereotypes. Whether autistic people’s direct lived experience with autism lends enhanced knowledge and reduced stigma through challenging deficit-based (mis)conceptions was not examined in prior work due to small sample sizes.

### Does a Focus on Normalizing Autism Contribute to Stigma toward Autism?

Consistent with the lack of consideration of how autistic individuals conceptualize autism, autistic adults continue to feel that their voices are not heard (Milton and Bracher, 2013; Pellicano et al., 2014b). Indeed, researchers express skepticism toward the prospect of yielding significant power to autistic people (Pellicano et al., 2014a). The continued disempowerment of autistic people is evident in the ongoing representation of autism in ways that many autistic people (and some parents of autistic people) disagree with strongly. Nicolaidis (2012), a physician and the mother of an autistic child, stated that recent autism awareness campaigns by researchers and funding agencies that describe autism as a kidnapper of children and as a living nightmare for families contribute to stigma toward autism. These campaigns are extreme examples of the medical model. A secondary aim of the current study was to investigate the hypothesis that medical-model orientations toward autism, such as the desire to normalize and cure autistic people, contribute to stigma toward autism.

### Aims and Hypotheses of the Current Study

We used an online survey to examine how autistic people and their close family members respond to commonly used measures of “scientific knowledge” about and stigma toward autism. This study builds upon a small but growing body of research using online surveys to compare how autistic and non-autistic people with diverse relationships to autism view how autism is represented and researched (Kapp et al., 2013; Pellicano et al., 2014a,b; Kenny et al., 2016; Fletcher-Watson et al., 2017) by administering measures that have been used with diverse types of **non-autistic people** internationally (e.g., Obeid et al., 2015; Harrison et al., 2017) to both autistic and non-autistic people with diverse relationships to autism.

This study was designed to assess the following hypotheses:

(1) Autistic adults and close family members of autistic people (non-mutually exclusive categories) would express more awareness of up-to-date scientific understanding about autism and less stigma toward autism when compared to people without each relationship to autism.
(2) Autistic adults would more commonly include internal experiences in their definitions of autism, define autism as a neutral difference, and/or critique the medical model than non-autistic people.
(3) Participants who endorsed more of a medical-model orientation toward autism, or expressed stronger interest in finding a cure for autism and in helping autistic people appear more normal, would endorse heightened stigma toward autism.

## Materials and Methods

### Recruitment

Approval was obtained from a university-based institutional review board prior to recruitment of participants to an anonymous online survey on SurveyMonkey. No compensation was given for participation. Online recruitment advertisements were posted on autism-related forums, weblogs, listservs, and groups on Facebook, Meetup, Reddit, Tumblr, and Twitter, as well as general online classified pages (Craigslist, Backpage, and Oodle), and were distributed through the social network of an autistic self-advocate (one of the researchers). Efforts were made to recruit participants from diverse sources representing different viewpoints on autism. Before entering the survey, all participants completed a consent form.

### Participants

Participants (*N* = 636) ranged in age from 18 to 73 years (*M* = 38.0; *SE* = 12.9; see **Tables 1**, **2**). Although 71.1% of participants were from the US, participants were recruited globally (76.5% North America, 12.0% Europe, 8.8% Australia, 1.1% South America, 0.9% Asia, and 0.6% Africa). The majority of participants identified as Caucasian/white (86.0%); others identified as mixed ethnicity (5.2%), Hispanic (3.6%), of Asian descent (2.8%), of African descent (1.9%), or Indigenous (0.5%). Participants self-identified non-mutually exclusive relationships to autism, including being autistic (*N* = 309) or not autistic (*N* = 327). Participants often reported multi-faceted relationships to autism. Therefore, classification as autistic was not mutually exclusive from other relationships to autism such as having an autistic nuclear family member (*N* = 360) or not having an autistic nuclear family member (*N* = 276).

**Table 1:** Characteristics of participants who identified as autistic or not autistic.

	Autistic	Not autistic	Group comparison
*N*	309	327	
Age*:* means (*SD*)	36.25 (13.60)	39.75 (11.96)	*p* = 0.001^∗^
RAADS-14 score:^1^ means (*SD*)	29.15 (8.67)	7.78 (10.03)	*p* < 0.001^∗^
Ethnicity: % Caucasian	87.1	85.0	*p* = 0.49
% Gender fluid	7.3	1.2	*p* < 0.001^∗^
% Female	62.7	84.6	*p* < 0.001^∗^
% Nuclear family member of autistic	55.7	57.5	*p* = 0.69
% Parent of autistic	25.9	49.5	*p* < 0.001^∗^
% Friend of autistic	49.5	26.3	*p* < 0.001^∗^
% Teacher of autistic	19.6	10.4	*p* = 0.001^∗^
% Live in USA	64.8	77.6	*p* = 0.001^∗^
% Have high school education or less	15.8	8.1	*p* = 0.003ˆ
% Have graduate degree or more	17.2	31.4	*p* < 0.001^∗^
% Professionals^2^	20.0	35.7	*p* < 0.001^∗^
% Technicians^2^	2.3	1.9	*p* = 0.78
% Unemployed^2^	42.7	31.9	*p* < 0.001^∗^


*^∗^Indicates that groups were significantly different; *p* ≤ 0.001; ˆ Indicates a non-significant trend; *p* ≤ 0.05. ^1^*N*s for this measure were lower as it was administered at the end of the survey so participants who chose not to “be trained” did not complete it: *N* Autistic = 231, *N* Not autistic = 252. ^2^ Open-ended responses to the question: “What is your current job or occupation? If you are not currently employed, please say so” were coded into 11 possible categories (including unemployment) according to the US Job classification guide: http://www.eeoc.gov/employers/eeo1survey/jobclassguide.cfm.*

**Table 2:** Characteristics of participants who identified as nuclear family members of autistic people or not.

	Nuclear family member	Not nuclear family	Group comparison
*N*	360	276	
Age: mean (*SD*)	41.18 (11.95)	33.92 (12.95)	*p* = 0.001^∗^
RAADS-14 score: mean (*SD*)^1^	18.83 (13.60)	16.94 (14.99)	*p* = 0.15
Ethnicity: % Caucasian	88.1	83.3	*p* = 0.11
% Gender fluid	3.4	5.1	*p* = 0.31
% Female	80.8	65.2	*p* < 0.001^∗^
% Parent of autistic	67.2	0	*p* < 0.001^∗^
% Friend of autistic	37.2	38.0	*p* = 0.87
% Teacher of autistic	13.6	17.0	*p* = 0.26
% Live in USA	68.6	74.9	*p* = 0.09
% High school or less	10.5	13.6	*p* = 0.26
% Graduate degree or more	25.0	23.8	*p* = 0.78
% Professionals^2^	27.2	29.2	*p* = 0.65
% Technicians^2^	1.4	3.0	*p* = 0.26
% Unemployed^2^	44.1	39.9	*p* = 0.29


*^∗^Indicates that groups were significantly different; *p* ≤ 0.001. ^1^*N*s for this measure were lower as it was administered at the end of the survey so participants who chose not to “be trained” did not complete it: *N* Nuclear family member = 270, *N* Not nuclear family = 213. ^2^ See **Table 1** for description of coding scheme.*

After assessing baseline conceptions of autism (the focus of this report), participants were asked to complete an online training based on extant research about autism. In order to assess baseline conceptions of autism among those who were disinterested in receiving autism training, all 636 participants who completed the pre-test were included in analyses reported here. Note that 75.9% (*N* = 483) of the participants completed the subsequent autism training, post-test and autism screener. Although the autism training is not the focus of this report, participation in the training was associated with increased knowledge about autism at post-test across groups, but no significant changes in stigma.

### Measures

Measures included a demographics questionnaire, a pre-test assessment of conceptions of autism, a training module (Gillespie-Lynch et al., 2015), a post-test mirroring the pre-test, and an autism screener. We embedded opportunities for participants to elaborate upon responses to each question if they wished to by asking: “Do you have anything else to say about this question?”

#### Demographic Survey

Participants were asked to indicate their gender, age, education, ethnicity, location, and occupation. Relationships to autism were assessed with this question: “Please select as many of the following types of relationships as you have had with people with autism spectrum disorders^2^: yourself, your child, your parent, your sibling, your spouse, your extended family member, your friend, your coworker, your student, your fellow student, your acquaintance, or other.” Participants were classified as autistic if they indicated that they were autistic and were classified as non-autistic if they did not indicate that they were autistic. Similarly, participants were classified as nuclear family members of autistic people if they reported having an autistic nuclear family member (child, parent, sibling, or spouse/romantic partner). Participants were classified as a teacher of an autistic person if they indicated that they had a student with autism. Participants were classified as a friend of an autistic person if they indicated having an autistic friend.

#### Autism Symptoms

The RAADS-14 (Eriksson et al., 2013) is a 14-item self-report measure (α = 0.92 in the current study) of autism symptoms. Scores range from 0 (never true) to 3 (true now and when I was young). A cutoff score of 14 or higher has strong sensitivity and adequate specificity in distinguishing between autism and other psychiatric disorders.

#### Pretest/Posttest

In addition to the measures described below, the researchers developed questions to assess conceptions of autism (see Appendix A).

##### Autism knowledge

Our primary assessment of autism knowledge was an adapted version of Stone’s Autism Awareness Survey (α = 0.66 in the current study; see Appendix B). Gillespie-Lynch et al. (2015) summarize how this survey has repeatedly been adapted to reflect changing research.

##### Autism stigma

We used an adapted version of the Social Distance Scale (Bogardus, 1933; see Appendix C). Six items (α = 0.83 in the current study) assessed willingness to engage with an autistic person at varying levels of intimacy (discussed in greater detail in Gillespie-Lynch et al., 2015).

### Analytic Approach

Descriptive analysis of the data indicated that the summed totals derived from the stigma and knowledge scales exhibited excessive kurtosis and/or skew. Although Wigley (2013) asserted that the sums of multiple Likert scale items that exhibit high internal consistency can be considered to reflect an interval scale of measurement, he acknowledged that sums of small numbers of Likert scale items (like our stigma measure) and/or scales with fairly low internal consistency (like our knowledge measure) retain an ordinal scale of measurement. Therefore, we used non-parametric analyses to evaluate all key study hypotheses. We evaluated conceptions of autism among those with each relationship to autism (e.g., self or family member) relative to those without that relationship rather than relative to those with a different relationship because participants often reported multiple overlapping relationships to autism, e.g., a participant could self-identify as autistic and also as the parent of an autistic person (see **Table 1**).

Given that autistic participants differed from non-autistic participants in terms of a number of demographic factors (i.e., age, country of residence, education, and gender) that have been associated with attitudes toward autism and other disabilities in prior work (e.g., Corrigan and Watson, 2007; Mak and Cheung, 2008; Obeid et al., 2015), we log transformed the data and conducted parametric replications of key non-parametric analyses to verify that key findings remained apparent after controlling for gender, country of residence (USA vs. not), education, age, and when more varied non-mutually exclusive binary relationships to autism (e.g., autistic vs. not, nuclear family member vs. not, teacher of autistic person vs. not, and friend of autistic person vs. not) were entered simultaneously into models. Although Norman (2010) asserted that parametric tests generate accurate conclusions even when used with highly skewed Likert scale data derived from small numbers of items, debates about this issue are unresolved (Wigley, 2013). Therefore, these follow-up parametric analyses should be considered only as evidence that our primary findings are not attributable to other characteristics besides relationships to autism.

As mentioned previously, the analyses in this report focus on the full sample (24.1% of whom did not complete the training and subsequent autism screener) in order to capture the perspectives of autistic people who were disinterested in receiving training. However, all findings except two (indicated with footnotes) remain significant if the sample is constrained to only those participants who completed the training and subsequent screener in order to compare autistic people who met the cutoff for likely autism on the screener to non-autistic people who were below the cutoff. Due to the large number of analyses conducted, we used Bonferroni corrections and only considered *p*-values ≤ 0.001 statistically significant; *p*-values ≤0.05 are reported as trends. Effect sizes are reported for all significant findings (King et al., 2010).

### Qualitative Coding

Transparency about how raw data are coded and interpreted allows readers to evaluate interpretations derived from qualitative analyses by situating examples within a broader context (Fujiura, 2015). Coding schemes were developed using thematic analysis guided by previous literature (e.g., Campbell et al., 2011; Kapp et al., 2013; Gillespie-Lynch et al., 2015) and emergent patterns in the data. Independent coders achieved reliability of 80% or greater across all coding categories, with 20% of the data coded independently for reliability.

Responses to the question “What are autism spectrum disorders in your own words?” were coded into non-mutually exclusive categories: Difference between communication and capacity (a mismatch between expressive and receptive communication, or atypical social communication), Social difficulties, RRIB, Internal (experiential definition including thinking, sensing, neurological), Opposition to the medical model (celebrate diversity and/or societal origin to challenges), Autism as “neutral difference” (atypical without valence), Support for medical model (autism as pathological and/or endorsing normalization), Confuse autism with intellectual disability, Other, and Don’t know.

Participants had the opportunity to provide open-ended elaborations in response to all survey questions. After reading all of the participants’ open-ended elaborations in response to all of the survey questions, we realized it was not feasible to qualitatively code *all* open-ended elaborations due to the very large number of open-ended elaborations. Therefore, we utilized a mixed-methods approach to select and code open-ended elaborations. We first identified the item from each category of questions about autism (i.e., importance of a cure/normalcy, stigma, and knowledge) that yielded the highest effect size when comparing autistic and non-autistic participants’ responses to the closed-ended question about that item (a quantitative measure of the questions that most clearly distinguished between autistic and non-autistic people) and then qualitatively coded open-ended elaborations in response to those questions. Elaborations were coded into non-mutually exclusive categories using thematic analysis. We report the proportion of autistic participants whose open-ended elaborations were classified into each coding category with illustrative examples of autistic participants’ responses.

## Results

### Group Differences in Baseline Knowledge and Stigma

#### Autism Knowledge

A Mann–Whitney *U* test revealed that autistic participants (*M* = 17.29, *SD* = 4.99) earned higher summed scores on the Autism Awareness Survey than non-autistic participants (*M* = 15.46, *SD* = 5.67, *Z* = –4.19; *p* < 0.001; *r* = 0.17). A non-significant trend toward nuclear family members exhibiting heightened knowledge relative to participants without an autistic nuclear family member was observed (*p* = 0.01). A linear regression predicting the log-transformed autism knowledge score, *F*(9,594) = 8.91, *R*^2^ = 0.13, *p* < 0.001, indicated that being autistic (*r*_p_ = –0.19; *p* < 0.001), having an autistic friend (*r*_p_ = –0.14; *p* = 0.001), and higher education (*r*_p_ = –0.18; *p* < 0.001) were all associated with greater knowledge. Non-significant trends toward heightened knowledge among women (*p* = 0.01), gender fluid participants (*p* = 0.04), and participants with an autistic nuclear family member were observed (*p*s = 0.02). Knowledge was not associated with having an autistic student (*p* = 0.49), living in the US (*p* = 0.08), or age (*p* = 0.59).

*Post hoc* Mann–Whitney *U* tests comparing autistic and non-autistic participants’ responses to the items comprising the Autism Awareness Survey revealed that autistic participants more strongly disagreed that autistic people can outgrow autism with treatment and that they are violent and more strongly agreed that autistic people have empathy than non-autistic people (see **Table 3**). Non-significant trends toward autistic people more strongly disagreeing that autistic people do not show attachments and more strongly agreeing that autistic people show affection and can grow up to go to college and marry were also observed.

**Table 3:** Autistic and not autistic participants’ average scores on autism awareness survey items.

	Autistic mean (*SD*)	Not Autistic Mean (*SD*)	Groupcomparison	Effect size
More Frequent Males	1.20 (1.20)	1.02 (1.33)	*p* = 0.10	*r* = 0.07
**Don’t show attachments**	-1.48 (0.90)	-1.27 (1.10)	*p* = 0.03ˆ	*r* = 0.08
**Deliberatively uncooperative**	-1.58 (0.77)	-1.53 (0.84)	*p* = 0.69	*r* = 0.02
Can go to college/marry	1.73 (0.75)	1.60 (0.89)	*p* = 0.006ˆ	*r* = 0.11
**One intervention for all**	-1.69 (0.87)	-1.69 (0.79)	*p* = 0.52	*r* = 0.03
**Proper treatment outgrow**	-1.61 (0.82)	-1.29 (0.97)	*p* < 0.001^∗^	*r* = 0.20
Show affection	1.54 (0.80)	1.42 (0.86)	*p* = 0.04ˆ	*r* = 0.08
**Most low IQ**	-1.50 (0.91)	-1.55 (0.82)	*p* = 0.81	*r* < 0.01
Lifelong disability	1.11 (1.29)	1.20 (1.02)	*p* = 0.54	*r* = 0.02
**Violent Tendencies**	-1.38 (0.93)	-1.02 (1.06)	*p* < 0.001^∗^	*r* = 0.18
**Disinterested friends**	-1.07 (1.05)	-1.01 (1.10)	*p* = 0.56	*r* = 0.02
Have empathy	1.41 (0.91)	0.85 (1.14)	*p* < 0.001^∗^	*r* = 0.27


*For bolded items, higher scores indicate *less* correct responses. ^∗^Indicates that groups were significantly different on Mann–Whitney *U* tests; *p* ≤ 0.001. ˆIndicates a non-significant trend; *p* ≤ 0.05.*

After indicating the degree to which they agreed that autistic people have empathy (this was the knowledge item that yielded the largest difference in effect size between autistic and non-autistic participants), 29% of autistic participants elaborated upon their responses. Among autistic participants who chose to elaborate about this question, 36% indicated that autism is associated with empathy overload (e.g., “excessive empathy without the ability to turn it off is part of the problem with being autistic”), 30% stated that autistic people have difficulty showing empathy (“Absolutely true… I am Autistic and I know lots of Autistics who, like me, have empathy. We just don’t transmit it to others in the typical way. If the questioner asking this disagrees, then the questioner needs to learn to question him or herself”), 26% indicated that their response depends on the type of empathy/situation (e.g., “it also depends on what we mean by empathy: compassion or mind-reading? We have compassion”), 20% described individual differences in empathy (“it depends on the person”), and 4% indicated that autism can be associated with reduced empathy (“not all of us do”).

Autistic participants also exhibited heightened awareness of recent changes in what constitutes autism knowledge (see Appendix A for the specific questions asked). Chi square tests revealed that autistic participants (61%) more frequently responded correctly when asked “how many autism spectrum disorders there are in the DSM-5?” by indicating that there is one autism spectrum disorder in the DSM-5 than non-autistic participants (40%), χ^2^ = 26.64, *p* < 0.001; φ = 0.20. Autistic participants (93%) also more frequently indicated that autism is hereditary than non-autistic participants (80%), χ^2^ = 21.07, *p* < 0.001; φ = 0.18. However, autistic participants (28%) less frequently acknowledged potential environmental causes of autism than their counterparts (53%), χ^2^ = 40.58, *p* < 0.001; φ = 0.25. Nuclear family members did not differ from others in their responses to these questions. A non-significant trend toward nuclear family members (89%) being more likely than others (84%) to say that autism is hereditary was observed, *p* = 0.05.

Autistic participants provided nuanced definitions of autism (see **Table 4** for examples); their definitions often simultaneously supported and opposed the medical model. Autistic participants were less likely to support the medical model than non-autistic participants (see **Table 5** for statistical results). They were more likely to oppose the medical model than others. Autistic participants were also more likely to describe autism as a neutral difference than others. Autistic participants’ definitions of autism were coded as internal more often than the definitions of non-autistic participants. Autistic participants were less likely to describe core social symptoms in their definitions than others.^3^ Instead, they more often highlighted a difference between communication and capacity relative to others.^4^ Nuclear family members’ definitions of autism did not differ from other participants’ across any of our coding categories. However, a non-significant trend toward family members (12%) more often highlighting a difference between communication and capacity relative to others (6%) was observed, *p* = 0.014.

**Table 4:** Examples of autistic participants’ definitions of autism spectrum disorders.

“A range of neurological phenotypes that affect how autistics perceive sensory input, language, social cues, etc. They also develop along a different neurodevelopmental trajectory; skills may be delayed or acquired in an atypical order.” ^I,M^
“A different way of perceiving and responding to the world and stimuli” ^I,N^
“I do NOT agree that the autism spectrum is a disorder. I take offense at the those who put together this survey calling AS a disorder. It is a DIFFERENCE. It is actually a more orderly wiring of the brain, so calling it a disorder is way off base. The extra nerve endings can be disabling. But when people on the spectrum find their hidden talent, they blossom and are a jewel to behold.” ^I,M,O^
“They are a nonsensical and arbitrary distinction within an obviously gradient set of traits, intended to define the autism continuum as a set of disorders.” ^O^


*Internal ^I^, Opposition to medical model ^O^, Autism as neutral difference ^N^, Support medical model^M^*

**Table 5:** Percentages of autistic and not autistic participants whose definitions of autism spectrum disorder received each qualitative code.

	Autistic	Not autistic	Group comparison	Effect size
Communication vs. capacity	13.9	5.2	*p* < 0.001^∗^	φ = 0.15
Social difficulties	37.5	54.1	*p* < 0.001^∗^	φ = 0.17
Restricted interests/behaviors	23.9	24.8	*p* = 0.85	φ< 0.01
Internal	68.9	54.7	*p* < 0.001^∗^	φ = 0.15
Oppose medical model	25.6	10.1	*p* < 0.001^∗^	φ = 0.20
Neutral difference	35.9	19.6	*p* < 0.001^∗^	φ = 0.18
Support medical model	54.7	76.5	*p* < 0.001^∗^	φ = 0.23
Confuse intellectual disability	0.6	0.9	*p* = 1.00	φ = 0.02
Don’t know	0.6	1.5	*p* = 0.45	φ = 0.04
Other	3.6	3.4	*p* = 1.00	φ< 0.01


*^∗^*p* ≤ 0.001 on chi-square tests.*

#### Stigma

A Mann–Whitney *U* test revealed that autistic participants (*M* = 7.23, *SD* = 2.20) reported lower stigma toward autism than non-autistic participants (*M* = 8.12, *SD* = 3.00), *Z* = –4.88; *p* < 0.001; *r* = 0.19. *Post hoc* Mann–Whitney tests revealed that differences in stigma between autistic and non-autistic participants were driven by autistic participants’ greater willingness to have an autistic person marry into the family and to marry one themselves (see **Table 6**).

**Table 6:** Autistic and not autistic participants’ average scores on items from the Social Distance Scale.

Willingness to engage with someonewith autism in the following ways:	Autistic mean (*SD*)	Not autistic mean (*SD*)	Group comparison	Effect size
Move next door	1.14 (0.39)	1.16 (0.48)	*p* = 0.87	*r* < 0.01
Spend an evening socializing	1.19 (0.48)	1.22 (0.49)	*p* = 0.21	*r* = 0.05
Collaborate with	1.26 (0.61)	1.29 (0.60)	*p* = 0.28	*r* = 0.04
Befriend	1.11 (0.36)	1.17 (0.47)	*p* = 0.14	*r* = 0.06
Have marry into family	1.16 (0.44)	1.39 (0.71)	*p* < 0.001^∗^	*r* = 0.18
Marry/date oneself	1.37 (0.76)	1.89 (1.03)	*p* < 0.001^∗^	*r* = 0.29


*Higher scores indicates greater stigma. ^∗^*p* ≤ 0.001 on Mann–Whitney *U* tests.*

Nuclear family members (*M* = 7.21, *SD* = 2.28) also reported lower stigma than participants without an autistic nuclear family member (*M* = 8.31, *SD* = 3.02), *Z* = –5.16; *p* < 0.001; *r* = 0.20. *Post hoc* Mann–Whitney tests revealed that nuclear family members of autistic people reported lower stigma than their counterparts for every item of the Social Distance Scale (see **Table 7**).

**Table 7:** Average scores on items from the Social Distance Scale of nuclear family members of autistic people and participants without an autistic nuclear family member.

Willingness to engage withsomeone with autism in thefollowing ways:	Nuclear family mean (*SD*)	Not nuclear family mean (*SD*)	Group comparison	Effect size
Move next door	1.11 (0.40)	1.21 (0.48)	*p* < 0.001^∗^	*r* = 0.14
Spend an evening socializing	1.12 (0.39)	1.32 (0.57)	*p* < 0.001^∗^	*r* = 0.23
Collaborate with	1.18 (0.50)	1.40 (0.70)	*p* < 0.001^∗^	*r* = 0.19
Befriend	1.09 (0.37)	1.21 (0.47)	*p* < 0.001^∗^	*r* = 0.20
Have marry into family	1.21 (0.53)	1.36 (0.68)	*p* = 0.001^∗^	*r* = 0.13
Marry/date oneself	1.50 (0.89)	1.82 (0.98)	*p* < 0.001^∗^	*r* = 0.19


*Higher scores indicates greater stigma. ^∗^*p* ≤ 0.001 on Mann–Whitney *U* tests.*

A linear regression predicting the log-transformed stigma sum, *F*(9,594) = 8.37, *R*^2^ = 0.11, *p* < 0.001, confirmed that being autistic (*r*_p_ = –0.17; *p* < 0.001) and having a nuclear family member who was autistic (*r*_p_ = –0.23; *p* < 0.001) were associated with lower stigma. Non-significant trends toward lower stigma among women (*p* = 0.002) and gender fluid participants (*p* = 0.02) were observed. Stigma was not associated with having an autistic friend (*p* = 0.22) or student (*p* = 0.76), living in the US (*p* = 0.57), age (*p* = 0.08), or education (*p* = 0.72).

After rating their willingness to marry someone autistic (the stigma item with the largest effect size), 30% of autistic participants elaborated upon their responses. Among autistic participants who chose to elaborate about the question, 38% of autistic participants’ responses described their prior or current relationships (e.g., “I am a person with ASD married to a person with ASD”), 24% indicated that they would prefer an autistic romantic partner (e.g., “I think I prefer it even though it’s tumultuous”), 20% indicated that they would prefer a non-autistic partner (e.g., “I don’t think I could ‘put up’ with another me”), 13% indicated that they were asexual (e.g., “Not interested; I’m a solitary, self-reliant person”), 13% indicated that relationships depend on the type of person (e.g., “I would marry or date them because I liked/loved them. Not just because they had autism. To me, my autism is like my hair color. No matter what I do, it’s a part of who I am. But, it’s just that. A part of the whole person. (All be it a rather large part)”), 7% indicated that the question was discriminatory (e.g., “This is so anti-autistic. Are you autistic yourself? Or do you just work for Autism Speaks!”), and 1% indicated that it depends on the type of autism (e.g., “depends on what their traits are. Some are very annoying.”).

### Group Differences in Attitudes toward Normalization

A Mann–Whitney *U* test revealed that autistic participants (*M* = -1.28, *SD* = 1.13) said it was less important to find a cure for autism relative to non-autistic participants (*M* = 0.20, *SD* = 1.55), *Z* = –11.98; *p* < 0.001; *r* = 0.48. Autistic participants (*M* = -0.88, *SD* = 1.30) also said it was less important to help autistic people appear normal (*M* = -0.15, *SD* = 1.41), *Z* = –6.61; *p* < 0.001; *r* = 0.26. Family members did not differ from others in the perceived importance of curing/normalizing autism.

After rating the perceived importance of finding a cure for autism, 51% of autistic participants elaborated upon their responses. Among autistic participants who chose to elaborate about the question, 87% provided neurodiversity-aligned responses (e.g., “Not just ‘not important.’ I think it’s detrimental to the lives of autistic people that so much money is put into finding a cure rather than developing support”), 60% indicated that autism is not a disease (“I don’t see autism as a ‘syndrome’ or ‘disease’ or ‘disorder’ that needs to be cured…, however if they came out with a ‘cure,’ I wouldn’t deny it to others, particularly those on the low-functioning end of the spectrum”), 15% indicated support for the medical model (e.g., “I think some people’s autism benefits them and they are content with being autistic but I personally struggle with it and it sometimes does not feel like my life with autism is worth living”), 14% indicated that autism is diverse (e.g., “I like how I am. I don’t necessarily like how I was treated. I do see how curing some forms of autism would be beneficial. I am referring to the forms where the person can’t move themselves around very well to communicate, but have normal intelligence and social understanding. As for autism forms like myself, I think I am fine in certain contexts and would not seek a cure”) and 12% critiqued the question/researcher (“HAHAHAHA Cure? Is this test from Autism Speaks?”).

### Do Attitudes toward Normalization Contribute to Stigma?

Spearman’s correlations revealed that heightened stigma toward autism was associated with more interest in curing autism among autistic participants, *r*_s_ (307) = 0.35, *p* < 0.001, in helping autistic people appear more normal, *r*_s_ (307) = 0.35, *p* < 0.001, and less knowledge of autism *r*_s_ (307) = –0.35, *p* < 0.001. Interest in a cure, *r*_s_ (325) = 0.35, *p* < 0.001, in helping autistic people appear more normal, *r*_s_ (325) = 0.34, *p* < 0.001, and less knowledge, *r*_s_ (325) = –0.38, *p* < 0.001, were also associated with heightened stigma among non-autistic participants.

A linear regression predicting log-transformed stigma in the entire sample, *F*(12,591) = 18.12, *R*^2^ = 0.27, *p* < 0.001, revealed that greater perceived importance of normalizing autistic people (*r*_p_ = 0.15; *p* < 0.001) and of curing autism (*r*_p_ = 0.15; *p* < 0.001), lower autism knowledge (*r*_p_ = –0.25; *p* < 0.001), and not having an autistic family member (*r*_p_ = –0.19; *p* < 0.001) were associated with heightened stigma. In this model, being autistic (*p* = 0.84), having an autistic friend (*p* = 0.58) or student (*p* = 0.85), education (*p* = 0.15), living in the US (*p* = 0.49), age (*p* = 0.33), being female (*p* = 0.07), and being gender fluid (*p* = 0.43) were not associated with stigma.

## Discussion

Findings generally support the notions that autistic people are autism experts through their lived experiences and reduced tendency to view autism through a deficit-defined medical model compared with non-autistic people. Autistic participants exhibited more knowledge about and less stigma toward autism, and more often described autism internally, or in terms of the lived experience of being autistic, than non-autistic people. They exhibited more awareness of recent changes in the diagnostic criteria for autism than non-autistic people. These findings suggest that autistic adults may be highly aware of diagnostic conceptions of autism, while often critical of their behavioral, deficit-only basis (Rosqvist, 2012a; Giles, 2014; Linton et al., 2014). Future research should compare autistic people’s understanding of autism to perspectives on autism expressed by autism researchers in order to evaluate the degree to which autistic people and autism researchers exhibit compatible forms of autism expertise.

Autistic participants’ conceptions of autism often aligned with those of the neurodiversity movement, in that they most frequently described autism as positive or neutral biological differences, and least frequently endorsed the medical model, e.g., by exhibiting the least interest in normalization or in finding a cure for autism. In our sample, less interest in normalizing autistic people and heightened knowledge of autism were associated with one another and with lower stigma toward autism. In addition to neurodiversity-aligned viewpoints, our findings suggest that other factors, such as experiences with autistic people, may reduce stigma toward autism. While autistic people reported far less interest in curing and normalizing autism than others, both autistic people and family members exhibited reduced stigma toward autism. Indeed, reduced interest in normalization and heightened knowledge about autism among autistic participants accounted for their reduced stigma toward autism.

A key implication of these findings is that interventions designed to normalize autistic people and cure-oriented organizations, legislation, and research may contribute to stigma toward autism. However, the current findings are correlational. Future research should examine if exposure to media messages indicating that autism should be cured and/or that autistic behaviors should be normalized leads to heightened stigma toward autism among autistic and non-autistic people.

These findings provide support for the importance of listening to autistic people and becoming more familiar with their experiences in order to address and counter stigma. Indeed, people aware of the neurodiversity movement are more likely to view autism as a positive identity that does not need a cure (Kapp et al., 2013). Although superficially surprising, our finding that numerically more autistic participants supported (55%), rather than opposed (26%), the medical model in their definitions of autism is consistent with prior research demonstrating overlap between the medical model and the neurodiversity movement in terms of shared recognition of challenges associated with autism (Kapp et al., 2013), which is consistent with a biopsychosocial model of autism (Kapp, 2013).

Findings offer clues about how stigmatizing misconceptions about autism might contribute to marginalizing autistic people in society. Autistic participants were more likely to recognize that most children cannot outgrow autism and to reject a misconceived autism-violence link. Media have repeatedly covered research on individuals who “lose” an autism diagnosis, such as a much-publicized newsmagazine article published during recruitment for this study that interviewed families of individuals who were described as having beaten autism (Padawer, 2014). Yet such individuals still have social difficulties (Orinstein et al., 2015). Autistic people often learn to cope by selectively masking autistic traits, but such learned behavior may be effortful and taxing, and does not mean a person is no longer autistic (American Psychiatric Association [APA], 2013). Similarly, empirical evidence has not found autistic people more likely to commit *any* crime (King and Murphy, 2014), yet media speculations about whether serial killers are autistic (Berryessa, 2014), and sympathy for parents who murder their autistic children (Waltz, 2008; Gross, 2012), may fuel exceptionally stigmatizing misperceptions of dangerousness (Feldman and Crandall, 2007).

Additionally, autistic adults were more likely to agree that autistic people have empathy; as autism by definition involves atypical social communication, autistic people may *express* their connections to others differently while legitimately feeling and sharing them. Consistent with emerging research (e.g., Smith, 2009), autistic participants frequently reported in their open-ended elaborations an excess of empathy that they struggled to express. Indeed, to successfully navigate “typical” society, autistic people may need to develop an explicit “theory of mind” even more than other people, with many autistic adults expressing considerable insights about their own and other minds (e.g., McGeer, 2004; Williams, 2004; DeNigris et al., unpublished). Non-autistic people’s tendency to view autistic people as lacking insight, aloof, prone to violence, and able to outgrow autism may lead them to doubt an individual’s autism diagnosis when that person is knowledgeable, outgoing, or otherwise effective in self-presentation (Smukler, 2005; Yergeau, 2010; Milton, 2014).

### Limitations and Future Directions

Reliance on a convenience sample of people who were willing and able to participate in an hour-long survey for no compensation limits the generalizability of findings. Participants were likely motivated to participate by an intrinsic interest in autism. Indeed, participants in this study exhibited substantially more knowledge of and less stigma toward autism than college students who participated in previous studies for academic credit (Gillespie-Lynch et al., 2015; Obeid et al., 2015).

Findings may not generalize to autistic participants who lack the verbal and computer skills needed to complete the survey. In addition, our analytic category of “nuclear family member of an autistic person” contains a great deal of unexamined variability as we could not distinguish between nuclear family members whose conceptions of autism were formed through relationships with autistic family members who do not speak and/or who have an intellectual disability and nuclear family members whose conceptions of autism were formed through relationships with highly verbal and/or gifted autistic people.

A fairly large number of participants in each group were unemployed, which suggests that our results (and online surveys more generally) might over-represent viewpoints of people who lack other things to do. Given that autistic people are more likely to engage with the neurodiversity movement online (Kapp et al., 2013), findings might represent the views of autism advocacy communities to a greater extent than samples of autistic participants recruited offline. Indeed, like autobiographies (Chamak et al., 2008; Davidson and Smith, 2009) and other online studies (e.g., Kapp et al., 2013; Pellicano et al., 2014b), this sample included a high proportion of autistic women and Caucasian individuals. Although we posted invitations to participate on a wide variety of Internet sites, including self-advocacy, anti-vaccine, and pro-cure groups, the degree to which the viewpoints captured are representative of the broader population remains unknown.

A key limitation of this study, and other Internet-based autism research (e.g., Kapp et al., 2013; Pellicano et al., 2014b; Kenny et al., 2016; Fletcher-Watson et al., 2017), is that we did not verify diagnosis of participants who self-identified as autistic. Some participants who self-identified as autistic might not have met criteria for autism while some participants who self-identified as not autistic may have been motivated to participate because they have heightened autistic traits, but have not been formally diagnosed. Many adults who meet criteria for autism may lack a formal diagnosis, if not also self-awareness, of autism (Brugha et al., 2011). Therefore, the current findings may not be representative of the viewpoints of many autistic people, including those who do not self-identify as autistic. Nevertheless, several factors complicate the ability to diagnose autism in adulthood. Behavioral assessments may lack sensitivity to coping mechanisms developed by adulthood; many autistic adults who no longer register on behavioral tests still demonstrate difficulties typical of autism, and self-report an autism diagnosis (Lai et al., 2011). Furthermore, many autistic adults lack reliable records of diagnostic history (Brugha et al., 2012). Women may be under-diagnosed, in part because autism may manifest differently in females (Kirkovski et al., 2013; Linton et al., 2014), who may tend to develop more superficial compensatory strategies by adulthood (Lai et al., 2011).

To address these limitations in generalizability, future research should be conducted online and offline using probability sampling and should include autistic participants with varied skills for whom diagnosis can be verified. Given the paucity of research comparing conceptions of autism across cultures (Norbury and Sparks, 2013), the inclusion of participants from around the globe was a potential strength of this study. However, more equal numbers of participants from different regions and representing different ethnicities than were obtained in the current study is needed to gain insights into how cultural differences may influence conceptions of autism.

## Conclusion

This study demonstrates that autistic people should be considered “autism experts” as they often build upon insights derived from the lived experience of being autistic by researching autism systematically. Autistic people who have developed heightened understanding of autism may be particularly well suited to teach other people about autism, as they tend to endorse less stigmatizing conceptions of autism, have reduced interest in making autistic people appear more normal, and may often have heightened empathy for the challenges others face (Komeda, 2015).

As our participants were adults who were motivated to seek out dialog about autism by participating in an uncompensated online study, we do not suggest that **all** autistic people exhibit heightened factual knowledge about or reduced stigma toward autism. As many of our survey respondents indicated, each person, regardless of whether or not they are autistic, is unique. Some autistic people seek out factual knowledge about autism while others believe that they can only be experts in their own particular form of autism (e.g., Jones et al., 2013). Autistic people have been reported to gain greater understanding of autism, themselves, and how to effectively educate others with age (Jones et al., 2015). The current findings suggest that autism trainings for autistic youth would benefit from inclusion of knowledgeable autistic adults as program mentors.

Findings also provide preliminary support for Nicolaidis’s (2012) hypothesis that autism awareness campaigns that focus on the importance of normalizing and curing autistic people do indeed contribute to stigma toward autism among both autistic and non-autistic people. Although knowledge is not yet power for many autistic people, identifying how autistic people think about autism is a first step toward developing research that is relevant to their interests and the needs of the community whom the research is intended to serve. Furthermore, the study suggests that involving autistic people as well as other people familiar with and knowledgeable about autism, such as close relatives, as empowered collaborators in the research process may help produce more accurate understanding of autism alongside greater acceptance and reduced stigma.

## Ethics Statement

The study has been approved by the Office for the Protection of Research Subjects; College of Staten Island; CUNY. Participants completed an IRB-approved online assent to participate in this study. Assent materials were approved by the IRB.

## Author Contributions

KG-L and SKK share primary authorship for this manuscript. KG-L developed the initial idea for this study and played a primary role in study design, analyses, the literature review and writing/revising of the manuscript. SKK played the primary role in study recruitment and a primary role in study design, qualitative analyses, the literature review and writing/revising of the manuscript. PB contributed to qualitative coding and the writing/editing of the manuscript. JP and BS contributed to study recruitment, qualitative coding, and editing the manuscript.

## Conflict of Interest Statement

The authors declare that the research was conducted in the absence of any commercial or financial relationships that could be construed as a potential conflict of interest.

## Footnotes

1 We use identity-first language to refer to autism, respecting the preference of many self-advocates (Kapp et al., 2013; Kenny et al., 2016)

2 We utilized the term autism spectrum disorder (ASD), or the overarching label for autism adopted in 2013 by the DSM-5, for this question and questions about the definition and diagnostic criteria for autism. We utilized the term “autism” for all other questions. A number of autistic participants indicated that the term ASD pathologizes autism. Therefore, we describe the questions we asked with the exact wording we used but use the terms “autism” or “autistic” throughout the rest of this report to respect the perspectives of autistic participants.

3 This was one of the two findings that were no longer statistically significant (*p* = 0.004) in the smaller sample of participants who completed the autism screener.

4 This was one of the two findings that were no longer statistically significant (*p* = 0.009) in the smaller sample of participants who completed the autism screener.
