# Supplementary material for: Whose Expertise Is It? Evidence for Autistic Adults as Critical Autism Experts
Source: Front Psychol. 2017 Mar 28;8:438. doi: 10.3389/fpsyg.2017.00438 (PMC5368186; doi:10.3389/fpsyg.2017.00438)
Supplement: Supplementary file 1 [file Appendix.docx]

Appendix A

Researcher Developed Measures of Conceptions of Autism

1. What are autism spectrum disorder(s) in your own words? Open-ended response

2. How many different autism spectrum disorders are there in the DSM-5?

Possible answers: 1, 3, 5, 10

3. What causes autism? Select as many choices as needed.

Possible answers: negative parenting, genes passed down from parents to children, new genes (mutations) in the genes of affected children, vaccines and environmental factors, such as toxins and pollutions

5. How important do you think it is to find a cure for autism?

6. How important do you think it is to help people with autism appear more normal?

Responses to questions 5-7 ranged from -2 (not important at all) to 2 (very important)

Appendix B

*Adapted Autism Awareness Survey*

Response choices included strongly disagree (-2), disagree (-1), neither agree nor disagree (0), agree (1), strongly agree (2).

1. Autism is more frequently diagnosed in males than females

**2. Children with autism do not show attachments, even to parents/caregivers**

**3. People with autism are deliberately uncooperative**

4. Children with autism can grow up to go to college and marry

**5. There is one intervention that works for all people with autism**

**6. With the proper treatment, most children diagnosed with autism eventually outgrow the disorder**

7. People with autism show affection

8. **Most people with autism have low intelligence**

9. Children with autism grow up to be adults with autism

**10. People with autism tend to be violent**

**11. People with autism are generally disinterested in making friends**

12. People with autism have empathy

Note: We added questions 10-12 to the adaptation developed by Gillespie-Lynch and colleagues (2015) and removed an item about the age at which autism is diagnosed after receiving feedback from clinicians that the age of reliable diagnosis remains unclear.

Bolded items are reverse scored.

Appendix C

Social Distance Scale

Response choices included definitely unwilling (4), somewhat unwilling (3), somewhat willing (2), definitely willing (1).

1) How willing would you be to move next door to someone with autism?

2) How willing would you be to spend an evening socializing with someone with autism?

3) How willing would you be to start a collaborative project with someone with autism?

4) How willing would you be to make friends with a person with autism?

5) How willing would you be to have a person with autism marry into the family?

6) How willing would you be to marry or date a person with autism?
